# Applicability and generalisability of the results of systematic reviews to public health practice and policy: a systematic review

**DOI:** 10.1186/1745-6215-11-20

**Published:** 2010-02-26

**Authors:** Nizar Ahmad, Isabelle Boutron, Agnès Dechartres, Pierre Durieux, Philippe Ravaud

**Affiliations:** 1 Centre d'épidémiologie Clinique, Hôpital Hôtel Dieu, AP-HP (Assistance Publique des Hôpitaux de Paris), 1 place du Parvis Notre-Dame, Paris 75181, France; 2 U738, INSERM, 1 place du Parvis Notre-Dame, Paris 75181, France; 3 Faculté de Médecine, Université Paris Descartes, 1 place du Parvis Notre-Dame, Paris 75181, France; 4 Centre de médecine fondée sur les preuves (EHESP, HAS, INSERM, APHP), 1 place du Parvis Notre-Dame, Paris 75181, France; 5 Santé Publique et Informatique Médicale, Université Paris Descartes, 15 rue de l'école de médecine, Paris 75006, France

## Abstract

**Background:**

The purpose of the study was to evaluate systematic reviews of research into two public health priorities, tobacco consumption and HIV infection, in terms of the reporting of data related to the applicability of trial results (i.e., whether the results of a trial can be reasonably applied or generalized to a definable group of patients in a particular setting in routine practice, also called external validity or generalisability).

**Methods:**

All systematic reviews of interventions aimed at reducing or stopping tobacco use and treating or preventing HIV infection published in the Cochrane database of systematic reviews and in journals indexed in MEDLINE between January 1997 and December 2007 were selected. We used a standardized data abstraction form to extract data related to applicability in terms of the context of the trial, (country, centres, settings), participants (recruitment, inclusion and exclusion criteria, baseline characteristics of participants such as age, sex, ethnicity, coexisting diseases or co-morbidities, and socioeconomic status), treatment (duration, intensity/dose of treatment, timing and delivery format), and the outcomes assessment from selected reviews.

**Results:**

A total of 98 systematic reviews were selected (57 Cochrane reviews and 41 non-Cochrane reviews); 49 evaluated interventions aimed at reducing or stopping tobacco use and 49 treating or preventing HIV infection. The setting of the individual studies was reported in 45 (46%) of the systematic reviews, the number of centres in 21 (21%), and the country where the trial took place in 62 (63%). Inclusion and exclusion criteria of the included studies were reported in 16 (16%) and 13 (13%) of the reviews, respectively. Baseline characteristics of participants in the included studies were described in 59 (60%) of the reviews. These characteristics concerned age in about half of the reviews, sex in 46 (47%), and ethnicity in 9 (9%).

Applicability of results was discussed in 13 (13%) of the systematic reviews. The reporting was better in systematic reviews by the Cochrane Collaboration than by non-Cochrane groups.

**Conclusions:**

Our study highlighted the lack of consideration of applicability of results in systematic reviews of research into 2 public health priorities: tobacco consumption and HIV infection.

## Background

Systematic reviews are an important source of valid evidence [1] because they identify, appraise and synthesize all the available evidence on a particular topic [1-3]. Theoretically, systematic reviews should evaluate and take into account the internal validity (i.e., the extent to which systematic errors or bias are avoided) of each trial included but also the applicability and generalizability or external validity (i.e., whether the results of a trial can be reasonably applied to a definable group of patients in a particular setting in routine practice)[4]. Several methodological works have been published to allow for adequately understanding and assessing internal validity [5-10]. Recently, the Cochrane Collaboration developed a specific tool to appraise the internal validity of trial results included in systematic reviews, the Risk of Bias tool [11] and research is still being conducted in this field[12]. In contrast, methodological research on the applicability of trial results is still at its beginning [13-15]. Some authors have highlighted that external validity and the applicability of trial research is a multi-dimensional concept [4,16]. They particularly noted that judging the external validity of study results is complex, consisting in prior knowledge, statistical considerations, and eligibility criteria [4]. Other authors focused on the reporting of applicability data to give readers sufficient information to be able to judge the external validity and applicability of the results of a trial [17,18]. For example, Glasziou and colleagues addressed this issue in stating that the description of interventions was insufficient to allow clinicians to replicate the intervention in clinical practice[19].

The main objective of this study was to evaluate systematic reviews of research into two public health priorities, tobacco consumption and HIV infection, in terms of the reporting of data related to the applicability of trial results (i.e., whether the results of a trial can be reasonably applied or generalized to a definable group of patients in a particular setting in routine practice, also called external validity or generalisability)[16].

A secondary objective was to compare the reporting of data related to applicability in systematic reviews published by the Cochrane Collaboration and other systematic reviews indexed in Medline [20].

## Methods

### Choice of the medical domain

We focused on 2 public health priorities: tobacco consumption and HIV infection[21]. Tobacco use is a leading preventable cause of death in the world; it is currently responsible for about 5 million deaths each year (one person every 6 sec). By 2030, the number of deaths will exceed 8 million a year [22,23]. The use of tobacco is on the increase in developing countries and among women in developed countries [22,24]. HIV/AIDS is also one of the most urgent threats to global public health. In 2007, the number of people living with HIV infection worldwide was estimated at 33.2 million; this number continues to increase, particularly in developing countries [25], where access to healthcare services is limited [26].

Managing smoking cessation as well as treating and preventing HIV infection relies on a combination of pharmacological treatments and behavioural interventions. The success of such treatments depends highly on patient characteristics and socioeconomic and cultural factors but also on the organization of healthcare. Therefore, systematic reviews of research into both topics must evaluate and consider the applicability of the results of that research.

### Search strategy

We identified all reports of systematic reviews of interventions aimed at reducing or stopping tobacco use and treating or preventing HIV infection that were published in the Cochrane database of systematic reviews and in journals indexed in MEDLINE between January 1 1997 and December 31 2007. We systematically searched MEDLINE for meta-analyses of articles and the Cochrane database of systematic reviews, looking for the following terms in the title, abstract and MeSH terms: "smoking cessation" OR "tobacco use cessation" OR "smoking reduction" OR "tobacco reduction" OR "smoking abstinence" OR "tobacco abstinence" for tobacco use and "HIV" OR "Human immunodeficiency virus" OR "AIDS" OR "acquired immunodeficiency syndrome" OR "sexually transmitted diseases" for HIV infection (see Additional file 1).

### Eligibility criteria and screening process

We collected the electronic records in an Endnote data file. Titles and abstracts of the electronic search results were screened by one of us (NA) to identify the relevant studies.

Using endnote search, we systematically search for reports having same authors, and the most recent review was included.

From selected abstracts, the full texts of articles were retrieved and reviewed by one of us (NA) to determine eligibility of studies for inclusion. For practical reasons, only one author performed the screening process. For quality assurance, another author (IB) double-checked the abstracts selected and the full-text articles excluded.

Reports were included if the study was identified as a systematic review of interventions aimed at stopping or reducing tobacco consumption or preventing or treating HIV infections.

A systematic review was defined as a scientific process seeking to collate all evidence that fits pre-specified eligibility criteria and to minimize bias by using explicit, systematic methods.

We excluded protocols of systematic reviews, systematic reviews focusing on a specific context (e.g., intervention for tobacco cessation in the dental setting), or a specific population (e.g., intervention for tobacco cessation during pregnancy or for hospitalized patients). In fact, because we focused on the adequate reporting of data related to the applicability of trial results, we decided not to include reviews of trials performed in a specific context or of specific patients such as the dental setting or hospitalized patients because evaluating the reporting of data related to the context or patient for these trials would be difficult if these criteria were eligibility criteria for the selected trial. Excluding these systematic reviews also allowed for a relatively homogeneous sample that should include reports containing all the applicability data domains. We also excluded systematic reviews concerning prevention or treatment of complications of HIV infection (e.g., opportunistic infections, Kaposi's sarcoma), and those evaluating a treatment for another disease among individuals with HIV infection (e.g., treatment of anemia in people with HIV). Overviews such as those published by Clinical Evidence were not selected in this study.

The systematic reviews were classified into 2 categories according to the data source: Cochrane reviews (i.e., systematic reviews performed and published by the Cochrane Collaboration) and non-Cochrane reviews (i.e., systematic reviews indexed in MEDLINE and performed by a non-Cochrane group).

### Data collection

#### Characteristics of the selected reports

We collected information on the category of treatment evaluated (i.e., pharmacological treatment such as oral drugs and nonpharmacological treatments such as education, quit lines or packages of care); for nonpharmacologic treatment, we determined whether the treatment was a therapist-dependent intervention (i.e., the success of the treatment depended on care providers' expertise and skill such as counselling, hypnosis, or acupuncture), the number of studies included, and the outcomes evaluated (e.g., for reviews of HIV infection: mortality; incidence of HIV infection; plasma HIV viral load; and for reviews of tobacco consumption: self-reported abstinence rate; self-reported smoking reduction rate; results of biological tests such as saliva, urine and serum nicotine levels; and expired carbon monoxide level). We checked whether a quantitative analysis (i.e., meta-analysis) was performed.

We determined whether and how the internal validity of the studies included in the systematic review was evaluated, reported and taken into account in the analysis and interpretation of the systematic review. We also recorded whether a narrative discussion or a summary description of the assessment of internal validity for the included studies was available.

#### Data related to applicability of results

To evaluate the reporting of data related to the applicability of trial results in systematic reviews, we developed a standardized data extraction form. To create this form, we relied on articles identified through a literature search [1,4,7,16,27-31] or known by or published by the authors of this article[17,18,32]. We also relied on the following reporting guidelines: the CONSORT Statement, the extension of the CONSORT Statement for nonpharmacologic treatment and the PRISMA Statement for reporting systematic reviewsand meta-analyses [31-33]. Our aim was not to perform a systematic review on this topic but rather to identify items deemed relevant. Using these articles, we generated a list of items deemed important: context of care, participants, intervention and outcome assessment.

Before data extraction, as a calibration exercise, the standardized form was tested by one of us (NA) on a separate set of 10 systematic reviews. One reviewer (NA) completed all the data extraction. A random sample of 30 articles was reviewed for quality assurance [34].

To evaluate the reporting of data related to applicability of results in the systematic review, we focused on 2 issues: 1) the key data related to applicability of results reported for each study included in the systematic review, and 2) the data related to applicability of results explored in the analysis and taken into account for the interpretation of the results.

For this purpose, in a first step, we checked whether the following data related to applicability were systematically reported in the review for each study included in the review: 1) the context of the trial: countries where the trial took place, the number of centres (because the applicability of a trial performed in only one centre could be questionable), and the setting (i.e., physicians, general medical hospital, university hospital); 2) participants: the method of recruitment (i.e., referral from physicians, self-selection of patients through advertisement), eligibility criteria, and essential data on baseline characteristics of participants (i.e., age, sex, ethnicity, coexisting diseases or co-morbidities, and socio-economic status); 3) treatment: the duration, intensity/dose of treatment, timing, delivery format and compliance of participants and the reporting of care providers' qualifications or expertise for reviews focusing on therapist-dependent interventions.

We systematically checked the systematic review's text, tables and appendices for descriptions of primary studies included in the systematic reviews. We particularly searched for the reporting of applicability data of primary studies. However, these data could be inconsistently reported. For example, a table describing each primary study could include the number of centres for some primary trials, but not for others. We hypothesized that this inconsistent reporting is probably related to these data being inconsistently reported in the published reports of primary trials. Consequently, the data are not reported in the table probably because they are not reported in the primary report.

We considered that if these data were reported for at least one primary study, they were systematically searched for by the reviewers.

In a second step, we checked whether applicability criteria were taken into account in the review analysis (e.g., subgroup analysis) and discussed the interpretation of the results as recommended by the PRISMA statement[35].

Unclear reporting was classified as being not reported, whereas partial reporting was classified as being reported.

### Statistical analysis

We used descriptive statistics; categorical variables were described with frequencies and percentages and quantitative variables with mean (SD). All data analysis involved use of SAS for Windows, Release 9.1 (SAS Institute, Cary, NC).

The data set is available in Additional file 2.

## Results

### Articles selected

**Figure 1:**
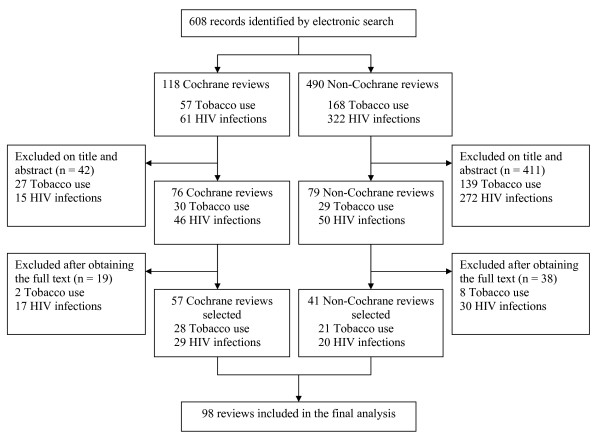
**Flow diagram of the selected systematic reviews**.

The flow of articles through the study is in Figure 1 and characteristics of the included systematic reviews are in Table 1. A total of 98 reviews were selected for the final analysis. The treatment evaluated concerned nonpharmacologic treatments in nearly two-thirds (n = 59) of the reports. The intervention evaluated was a therapist-dependent intervention in 45 (46%) reviews. Quantitative analyses were performed in 79 (81%) of the systematic reviews. In the field of tobacco use, 42 (86%) of the systematic reviews selected only randomised controlled trials, whereas in the field of HIV infection, more than half of the systematic reviews selected only randomised controlled trials. For reviews of tobacco use, the outcome of the review was abstinence in 37 (75%) reports. For those of HIV, 22 (44%) had at least one clinically relevant primary outcome (e.g., mortality or incidence of HIV infection).

**Table 1:** Characteristics of the selected systematic reviews

	All reviews N = 98	Cochrane reviews N = 57	Non-Cochrane reviews N = 41	Tobacco use N = 49	HIV infection N = 49
**Type of treatment, N (%)**					
Pharmacological treatment	33 (34)	23 (40)	10 (24)	17 (35)	16 (33)
Nonpharmacological treatment	59 (60)	28 (49)	31 (76)	28 (57)	31 (63)
Both treatments	6 (6)	6 (10)	0	4 (8)	2 (4)
**Therapist-dependent intervention, N (%)**	45 (46)	25 (44)	20 (49)	23 (47)	22 (45)
**Method of the review, N (%)**					
Quantitative analysis	79 (81)	44 (77)	35 (85)	40 (82)	39 (79.6)
**Number of studies included, median (Q1-Q3)**	14 (7-25)	9 (5-21)	18 (12-27)	14 (7-26)	14 (7-20)
**Trial design, N (%)**					
Randomised controlled trials (RCTs) only	68 (69)	46 (81)	22 (54)	42 (86)	26 (53)
RCTs + nonrandomized trials	24 (24)	8 (14)	16 (39)	5 (10)	19 (39)
Nonrandomized trials only	6 (6)	3 (5)	3 (7)	2 (4)	4 (8)
**Reporting of key data to describe included studies in a table or figure, N (%)**	87 (88)	53 (93)	33 (88)	40 (82)	46 (94)
**Internal validity assessment, N (%)**	71 (72)	53 (91)	18 (44)	35 (86)	36 (73)
Quality score	11 (11)	1 (2)	10 (24)	10 (20)	1 (2)
Allocation sequence generation	44 (52)	37 (65)	7 (17)	30 (61)	14 (29)
Allocation concealment	59 (58)	51 (89)	8 (19)	31 (63)	28 (57)
Blinding of participants	21 (21)	17 (30)	4 (10)	6 (12)	15 (31)
Blinding of care providers	22 (22)	18 (32)	4 (10)	5 (10)	17 (35)
Blinding of outcome assessors	10 (10)	6 (10)	4 (15)	4 (12)	6 (12)
Intent-to-treat analysis	13 (13)	8 (14)	5 (12)	5 (10)	8 (16)
**Method to account for internal validity, N (%)**					
Sensitivity analysis	8 (8)	3 (5)	5 (12)	4 (8)	4 (8)
Weighting factor	0	0	0	0	0
Meta regression	0	0	0	0	0
Narrative discussion of internal validity	52 (52)	48 (84)	4 (10)	23 (47)	26 (53)

Q1-Q3 = interquartile range 1 to 3

The internal validity of the studies included in the systematic review was assessed in 71 (72%) of the reviews; 53 (91%) of the Cochrane reviews and 18 (44%) of the non-Cochrane reviews. Most reviews provided a narrative discussion of the assessment of internal validity of the trials included but did not specifically analyze the assessments of internal validity in the analysis.

### Data related to applicability of results

The reporting of applicability data for each study included in the systematic reviews is described in Table 2. The setting was systematically considered in 45 (46%) of the reviews, the number of centres in 21 (21%) and the country where the trial took place in 62 (63%).

**Table 2:** Data related to applicability of results systematically reported for trials included in the systematic reviews

	All reviews N = 98	Cochrane reviews N = 57	Non-Cochrane reviews N = 41	Tobacco use N = 49	HIV infection N = 49
**Setting and centres**					
Setting of recruitment	45 (46)	33 (58)	12 (29)	24 (49)	21 (43)
Number of centres	21 (21)	19 (33)	2 (5)	15 (31)	6 (12)
Country where the study took place	62 (63)	41 (72)	21 (51)	29 (59)	33 (67)
**Recruitment**					
Method	18 (18)	18 (32)	0	15 (31)	3 (6)
**Participants**					
Inclusion criteria	16 (16)	13 (23)	3 (7)	5 (10)	11 (22)
Exclusion criteria	13 (13)	11 (19)	2 (5)	4 (8)	9 (18)
Baseline characteristics of participants	59 (60)	42 (74)	17 (41)	29 (59)	30 (61)
Age	47 (48)	35 (61)	12 (29)	26 (53)	21 (43)
Mean or median only	39 (40)	29 (51)	10 (24)	21 (43)	18 (37)
Mean (SD) or median (IQR)	3 (3)	3 (5)	0	0	3 (6)
Minimum and maximum value only	17 (17)	12 (21)	5 (12)	8 (16)	9 (18)
Sex	46 (47)	33 (58)	13 (32)	22 (45)	24 (49)
Ethnicity	9 (9)	4 (3)	5 (12)	2 (4)	7 (14)
Socioeconomic status	1 (1)	1 (2.0)	0	0	1 (2)
Co-morbidities	0	0	0	0	0
Associated treatments	0	0	0	0	0
**Treatment**					
Duration of treatment	61 (62)	40 (70)	21 (51)	35 (71)	26 (53)
Intensity/dose of treatment	64 (65)	44 (77)	20 (49)	34 (69)	30 (61)
Delivery format of treatment	72 (73)	49 (86)	23 (56)	37 (75)	35 (71)
Treatment timing	52 (53)	40 (70)	12 (29)	30 (61)	22 (45)
Compliance of participants	3 (3)	0	3 (7)	0	3 (6)
**Outcome assessment**					
Primary outcome	67 (68)	52 (91)	15 (37)	22 (45)	35 (71)
Adverse events	5 (5)	3 (5)	2 (5)	2 (4)	3 (6)
**Follow up**					
Length of follow-up	34 (35)	17 (30)	17 (41)	20 (41)	14 (29)
Number of visits	13 (13)	12 (21)	1 (2)	9 (18)	4 (8)

Inclusion criteria for each included study were considered in 16 (16%) reviews (23% for Cochrane and 7% for non-Cochrane reviews) and exclusion criteria in 13 (13%) (19% for Cochrane and 5% for non-Cochrane reviews). Baseline characteristics of participants were described in 59 (60%) of the reviews, with important data such as age, sex, ethnicity, and socioeconomic status frequently missing. Important criteria to reproduce the intervention, such as treatment duration, dosage or intensity, delivery format, and timing were missing in one-quarter to one-half of the reviews. Information related to care providers' qualifications and specific training were reported in 7 (7%) and 2 (2%) reviews, respectively. The primary outcomes of each included study were systematically reported in 67 (68%) reviews, length of follow-up was missing in 64 (65%) of the reports, and adverse effects were reported in only 5 (5%) reviews. The reporting of some of these data (setting, number of centres, method of recruitment, baseline characteristics, delivery format of treatment and primary outcome) was better for Cochrane reviews than for non-Cochrane reviews. For example, in a systematic review on acupuncture and related interventions for smoking cessation[36], the authors gave a description of all trials included in the review in a table, indicating for each trial (if it was reported in the primary trial) the country where the trial took place, the mode of recruitment, patients' eligibility criteria, and details of the intervention (number of sessions, duration of each session, acupuncture points etc.).

To evaluate the influence of applicability criteria, analyses were stratified by centre, country or setting in 6 (6%) systematic reviews, by components of intervention in 41 (42%) reviews and by characteristics of participants in 7 (7%) systematic reviews. Applicability was discussed in the discussion section of 13 (13%) reviews.

## Discussion

This study assessed the methods and reporting of information on the applicability of trial results in systematic reviews that might aid in applying their results. We assessed 98 systematic reviews of research into interventions aimed at reducing or stopping tobacco use and treating or preventing HIV infection published during a recent 10-year period. The applicability of results was poorly reported and taken into account in these systematic reviews.

These results and our finding of lack of information on cultural and socioeconomic contexts, patient characteristics, and the content of the interventions in the reviews questions how decision makers and clinicians can use the results of such reviews [16,19,29,37-39]? This situation is particularly problematic in the fields we studied because more than half of the interventions concerned nonpharmacologic treatments such as behavioural interventions, which are complex and difficult to reproduce in clinical practice, and the socioeconomic and cultural contexts are important for their success in clinical practice.

Considering that applicability is essential for the developers of guidelines to grade the strength of recommendations, the Grading of Recommendations Assessment, Development and Evaluation (GRADE) system for grading the evidence of clinical guidelines clearly tackles this issue [40].

This grading system separates decisions regarding the quality of evidence (mainly considering the internal validity of the studies) from strength of recommendations (i.e., taking into account the risk-benefit balance). The strength of the recommendations are likely to differ by practice settings or patient group[41]. For example, in the field of cardiovascular risk management, randomized controlled trial-based evidence was downgraded most often because of reservations about the applicability of the trial results[42].

Most of the effort of methodological research in the field of systematic reviews, particularly the work by the Cochrane Collaboration, has focused on the evaluation of internal validity. The results of these efforts emphasize a better consideration of internal validity in systematic reviews performed by the Cochrane Collaboration [43,44]. However, evaluating applicability of results is of similar importance. In the field of HIV interventions for example, Merson et al. highlighted "the lack of [...] contextual data to tailor specific interventions is reprehensible, particularly in view of the large amount of resources that have been invested to date in HIV prevention efforts, and hinders policy makers' ability to make informed decisions on prevention priorities"[45]. The Applicability and Recommendations Methods Group (ARMG) of the Cochrane Collaboration is nevertheless tackling this important but difficult issue, and recommendations are pending. To add to this discussion, from our results, we propose 3 recommendations for performing systematic reviews and 3 recommendations for methodological research (see Additional file 3).

Assessing applicability and external validity is difficult[4]. As well, deciding which items are relevant and should be reported is difficult. Further, the importance of some items may vary by context (e.g., assessing pharmacologic treatments or nonpharmacologic treatments). Therefore, when planning a systematic review, the protocol should define which applicability items are important and should be collected and reported. Not all of the applicability items we evaluated necessarily interact with effect size. However, methodological work evaluating the impact of applicability on effect size is lacking, and therefore, making a definitive statement on this issue is difficult. Further, even if some applicability items do not interact with effect size, details of applicability items must be provided to allow clinicians, patients and decision makers decide whether and how they will apply the results in clinical practice. Items identified as possibly interacting with treatment effect estimates should be offered as *a priori *explanations of heterogeneity, and an exploration of whether treatment differs across these characteristics should be undertaken. Other items aimed at helping readers appraise the applicability of the trials in their context should be reported. Online addenda now provide a great opportunity to adequately describe the included studies for interested readers without burdening every reader.

The reporting of data related to external validity is now clearly indicated in the PRISMA statement for reporting systematic reviews and meta-analyses. The PRISMA statement clearly focuses on the need to consider components to frame the question known by the acronym "PICOS" (Patient, Intervention, Comparator group, Outcome, and Study design). Focusing on PICOS, the statement should improve the reporting of external validity. In fact, issues related to PICOS affect several PRISMA items with the need to clearly describe participants, the disease, the setting of care, the intervention, and the comparator.

One explanation for the differences between Cochrane and non-Cochrane reviews could be linked to the space constraints (limited word count, number of tables and figures) requested by some editors but not by publishers of Cochrane Library reports. Further, the question evaluated in Cochrane and non-Cochrane reviews differed in terms of the type of treatment (pharmacologic or nonpharmacologic); for example, about half of the Cochrane reviews evaluated drugs, whereas non-Cochrane reviews more often evaluated nonpharmacological treatments

This study has several limitations. First, we focused on two medical areas, and these results should be confirmed in other medical areas. However, we chose tobacco consumption and HIV infection because they are among the first 5 causes of mortality in the world. Second, currently no consensus exists on how to assess the applicability of study results, we identified the applicability items following a literature review, and the relevance of some items might vary. Third, we did not consider the importance of each item even though it may vary according to context (e.g., assessing pharmacologic treatments or nonpharmacologic treatments). Fourth, during the appraisal process, we assumed that if data were reported in at least one randomized controlled trial included on the systematic review, this data had been gathered systematically in the systematic review. This assumption may have overestimated the reporting. Fifth, we excluded systematic reviews of reports for specific contexts or a specific population, which may have biased our sample of reviews to those widely applicable.

Finally, the screening process and the data collection were performed by only one reviewer. However, a quality assurance procedure was performed.

## Conclusions

In conclusion, despite the large number of systematic reviews published, our study highlights the lack of consideration of data related to the applicability and generalisability of results in these reviews.

## Abbreviations

HIV: Human Immunodeficiency Virus; AIDS: Acquired Immunodeficiency Syndrome; PICO: Patient-Intervention-Comparison-Outcome; CONSORT: Consolidation of the standards of reporting trials; PRISMA: Preferred Reporting Items for Systematic Reviews and Meta-Analyses; GRADE: Grading of Recommendations Assessment, Development and Evaluation; ARMG: Applicability and Recommendations Methods Group; RCTs: Randomised controlled trials.

## Competing interests

The authors declare that they have no competing interests.

## Authors' Contributions

NA participated in the study concept and design, in the literature search and identifying relevant systematic reviews, in the acquisition of data from included reviews, performed the statistical analysis, participated in the analysis and interpretation of data and drafted the manuscript. IB participated in the study concept and design, in the literature search and identifying relevant systematic reviews, in the analysis and interpretation of data, in the critical revision of manuscript for important intellectual content and in the study supervision. AD participated in the acquisition of data from included reviews and in the critical revision of manuscript for important intellectual content. PD participated in the analysis and interpretation of data and in the critical revision of manuscript for important intellectual content. PR participated in the study concept and design, in the analysis and interpretation of data, in the critical revision of manuscript for important intellectual content and supervised the study. NA is guarantor and has full access to all of the data in the study and takes responsibility for the integrity of the data and the accuracy of the data analysis.

## Supplementary Material

Additional file 1 **Appendix 1**. Search strategy Click here for file

Additional file 2 **Dataset**. Data extracted from included reviews Click here for file

Additional file 3 **Appendix 2**. Implications of the study results Click here for file
