# Supplementary material for: Applicability and generalisability of the results of systematic reviews to public health practice and policy: a systematic review
Source: Trials. 2010 Feb 26;11:20. doi: 10.1186/1745-6215-11-20 (PMC2838881; doi:10.1186/1745-6215-11-20)
Supplement: Additional file 1 — Appendix 1. Search strategy [file 1745-6215-11-20-S1.DOCX]

**Appendix 1: Search strategy**

PubMed search strategy

Tobacco use

#1:

(smoking [Title/Abstract] AND cessation[Title/Abstract]) OR (tobacco[Title/Abstract] AND cessation[Title/Abstract]) OR (smoking[Title/Abstract] AND reduction[Title/Abstract]) OR (tobacco[Title/Abstract] AND reduction[Title/Abstract]) OR (smoking[Title/Abstract] AND abstinence[Title/Abstract]) OR (tobacco[Title/Abstract] AND abstinence[Title/Abstract]) AND (Meta-Analysis[ptyp] AND ("1997/01/01"[PDAT] : "2007/12/31"[PDAT]))

#2:

(smoking cessation [MeSH Terms]) OR (tobacco use cessation [MeSH Terms]) OR (smoking reduction [MeSH Terms]) OR (tobacco reduction [MeSH Terms]) OR (smoking abstinence [MeSH Terms]) OR (tobacco abstinence [MeSH Terms]) AND (Meta-Analysis[ptyp] AND ("1997/01/01"[PDAT] : "2007/12/31"[PDAT]))

Final search:

#1 OR #2 AND (Meta-Analysis [ptyp] AND ("1997/01/01"[PDAT] : "2007/12/31"[PDAT]))

HIV infection

#3:

HIV[Title/Abstract] OR (Human[Title/Abstract] AND immunodeficiency[Title/Abstract] AND virus[Title/Abstract]) OR AIDS[Title/Abstract] OR (acquired[Title/Abstract] AND immunodeficiency[Title/Abstract] AND syndrome[Title/Abstract]) OR (sexually[Title/Abstract] AND transmitted[Title/Abstract] AND diseases[Title/Abstract]) AND (Meta-Analysis[ptyp] AND ("1997/01/01"[PDAT] : "2007/12/31"[PDAT]))

#4:

"hiv"[MeSH Terms] OR "acquired immunodeficiency syndrome"[MeSH Terms] OR "sexually transmitted diseases"[MeSH Terms] AND (Meta-Analysis[ptyp] AND ("1997/01/01"[PDAT] : "2007/12/31"[PDAT]))

Final search:

#3 OR #4 AND (Meta-Analysis[ptyp] AND ("1997/01/01"[PDAT] : "2007/12/31"[PDAT]))

**Cochrane research strategy:**

Tobacco use

"(smoking cessation in Title, Abstract or Keywords) or (tobacco use cessation in Title, Abstract or Keywords) or (smoking reduction in Title, Abstract or Keywords) or (tobacco reduction in Title, Abstract or Keywords) or (smoking abstinence in Title, Abstract or Keywords) or (tobacco abstinence in Title, Abstract or Keywords) in Cochrane Database of Systematic Reviews of type review"

HIV infection

"(HIV in Title, Abstract or Keywords) or (Human immunodeficiency virus in Title, Abstract or Keywords) or (AIDS in Title, Abstract or Keywords) or (acquired immunodeficiency syndrome in Title, Abstract or Keywords) or (sexually transmitted diseases in Title, Abstract or Keywords), from 1997 to 2007 in Cochrane Database of Systematic Reviews of type review"
