# Supplementary material for: Applicability and generalisability of the results of systematic reviews to public health practice and policy: a systematic review
Source: Trials. 2010 Feb 26;11:20. doi: 10.1186/1745-6215-11-20 (PMC2838881; doi:10.1186/1745-6215-11-20)
Supplement: Additional file 3 — Appendix 2. Implications of the study results [file 1745-6215-11-20-S3.DOCX]

**Appendix 2: Implications of the study results**

**IMPLICATIONS FOR methodological RESEARCH**

Research is needed on the following:

1. Identifying essential items related to applicability by type of treatment.
2. How to report applicability across trials. For example, what is the best way to provide information on age (range of mean ages across the trials, weighted average, etc.)
3. How to interpret the applicability of the results of a systematic review.

**Implications for peforming systematic reviews**

1. In the protocol for systematic reviews, authors must identify which applicability items are important (according to the type of treatment evaluated) for the systematic review and should be collected and reported.
2. In the reports of systematic reviews, the reporting of applicability items should be standardized and complete for each primary trial included in the systematic review (e.g., in a table). The report should clearly specify when the applicability item is unclear.
3. Applicability should be systematically discussed in the systematic review and clearly indicated in the conclusions of the systematic review.
